# Leaf Gas Exchange and Fluorescence of Two Winter Wheat Varieties in Response to Drought Stress and Nitrogen Supply

**DOI:** 10.1371/journal.pone.0165733

**Published:** 2016-11-01

**Authors:** Xiubo Wang, Lifang Wang, Zhouping Shangguan

**Affiliations:** State Key Laboratory of Soil Erosion and Dryland Farming on the Loess Plateau, Institute of Soil and Water Conservation, Chinese Academy of Sciences and Ministry of Water Resources, Yangling, Shaanxi, 712100, P.R. China; University of Delhi, INDIA

## Abstract

Water and nitrogen supply are the two primary factors limiting productivity of wheat (*Triticum aestivum* L.). In our study, two winter wheat varieties, Xinong 979 and large-spike wheat, were evaluated for their physiological responses to different levels of nitrogen and water status during their seedling stage grown in a phytotron. Our results indicated that drought stress greatly reduced the net photosynthetic rate (*P*_*n*_), transpiration rate (*E*), and stomatal conductance (*G*_*s*_), but with a greater increase in instantaneous water use efficiency (WUE). At the meantime, the nitrogen (N) supply improved photosynthetic efficiency under water deficit. Parameters inferred from chlorophyll *a* measurements, i.e., photochemical quenching coefficient (qP), the maximum photochemical efficiency (F_v_/F_m_), the quantum yield of photosystemII(Φ_PSII_), and the apparent photosynthetic electron transport rate (ETR) decreased under water stress at all nitrogen levels and declined in N-deficient plants. The root–shoot ratio (R/S) increased slightly with water stress at a low N level; the smallest root–shoot ratio was found at a high N level and moderate drought stress treatment. These results suggest that an appropriate nitrogen supply may be necessary to enhance drought resistance in wheat by improving photosynthetic efficiency and relieving photoinhibition under drought stress. However, an excessive N supply had no effect on drought resistance, which even showed an adverse effect on plant growth. Comparing the two cultivars, Xinong 979 has a stronger drought resistance compared with large-spike wheat under N deficiency.

## Introduction

Wheat (*Triticum aestivum* L.) is the most widely distributed cereal crop in the world. In many agricultural areas, especially northern China, drought and nitrogen deficiency are two major limiting environmental factors to photosynthesis and plant growth [1–3]. Several studies have shown that drought stress strongly affects growth and nitrogen metabolism [3]. Nitrogen application can contribute to drought resistance to a certain extent in some plants, such as cotton and *Brassica carinata* [4, 5]. In winter wheat, the responses of nitrogen supply on leaf photosynthesis gas exchange and water use efficiency were also variable under different water status [6].

Photosynthesis is the most important source of biomass accumulation in all plants, algae and cyanobacteria, and it is one of the most sensitive physiological processes to abiotic stress [7]. The photosynthetic rate, the transpiration rate, and the stomatal behavior are changed in varying degrees when plants are subjected to drought and N stresses. The effects of drought stress on photosynthesis can be divided into stomatal limitation and non-stomatal limitation [8, 9]. It is generally acknowledged that reducing CO_2_ diffusion from the atmosphere to the site of carboxylation due to stomatal closure and reduced mesophyll conductance, which in turn, contributes to a decrease in photosynthesis under water stress conditions [9, 10]. Nitrogen is an important component in the synthesis of plant pigments and photosynthetic enzymes in plants, which directly or indirectly affects photosynthesis of crops [11]. Nitrogen could enhance the stomatal regulation of plants, and also well maintain the physiological function of photosynthetic apparatus by increasing chlorophyll content, photosynthetic oxygen evolution rate and the light saturation point [12]. Drought and nitrogen deficiency can significantly reduce the net photosynthetic rate of plants and ribulose bisphosphate carboxylase oxygenase (Rubisco) activity, but drought did not affect Rubisco activity under sufficient nitrogen supply [13]. Nitrogen deficiency increased a strong sensitivity of stomata to drought.

Chlorophyll *a* fluorescence measurement is a diagnostic technique to indirectly, but, in a non-invasive manner detect photosynthetic reactions in plants and the tolerance to environmental stresses, which can be used to effectively analyze the effects of abiotic stress factors on photosynthesis [14]. Chlorophyll *a* fluorescence measurements could be widely used to examine photosynthetic performance in leaves in laboratory and controlled environment. It can provide useful information on physical changes in pigment-protein complexes, excitation energy transfer, primary photochemistry and the operating quantum efficiency of electron transport through PSII[15, 16]. Drought stress can not only cause structural damage to PSII and light-harvesting complexes directly, but also affect the process of photosynthetic electron transport and photophosphorylation [17]. The photosynthetic pigment molecules in energy absorption, transfer and conversion are mostly composed of proteins, Therefore, nitrogen deficiency leads to the decrease of the content and function of PSI and PSII, which affect the conversion of photochemical energy. Nitrogen plays an important role in transfer and dissipation of excess light energy, which can relieve damage of excess excitation energy to photosynthetic apparatus and keep the PSII maximum photochemical efficiency (F_v_/F_m_) at a higher level [18].

Water and nutrient are two coupled physiological processes that interact with each other [19]. Hence, studies on the drought, nitrogen or their interactions on plant morphological and physiological responses are important for future studies. Many researchers have investigated the responding mechanism of water regime and nitrogen supply on the yield of wheat plants [20, 21]. The effects of water status and nitrogen application on leaf gas exchange have also been measured [22–24]. However, the research results have not been consistent due to differences in the planting area, experimental design, varieties and fertilizer levels.

In this study, seedlings of the two wheat varieties with different water adaptability were subjected to drought for 7 days under different levels of nitrogen supply. Photosynthesis and chlorophyll *a* fluorescence were measured to investigate wheat drought tolerance. The main objectives of the study were to (1) investigate the responses of gas exchange and chlorophyll *a* fluorescence to various water stress and nitrogen supply conditions and the relationship of these traits with biomass; (2) determine whether the genotypic variability in photosynthesis and chlorophyll *a* florescence of wheat is affected by water and nitrogen. Our results helped us in finding differences in photosynthetic acclimation mechanisms between the two varieties under drought stress. We believe that these can be used to explore the mechanism of water and fertilizer coupling, and provide scientific basis for improving the wheat yield in response to fluctuations in environmental conditions.

## Materials and Methods

### Plant materials and Experimental design

Wheat seeds (*Triticum aestivum* L. cv. Xinong 979; *Triticum aestivum* L. cv. Large-spike wheat) were provided by Northwest Agriculture and Forestry University (Yangling Shaanxi, China). Large-spike wheat was chosen from high-yield, large-spike lines of wheat, which had superior physiological characteristics and high yield potential [25]. Xinong 979, a major winter wheat cultivar planted in the southern area of the Huang-Huai wheat region in China, was also used in this study.

Healthy seeds were disinfected with 0.1% (w/v) HgCl_2_ for 5 min, then rinsed with deionized water for 5 min, and kept in water for 24h. Seeds were placed on sterile filter paper in a incubator under a constant temperature of 25°C and transplanted in quartz sand after germination. When the wheat seedlings grew to leaf stage 2, they were transplanted to an opaque plastic basin with 20 cm in diameter and 28 cm in height, and planted on a foam board. All seedlings were grown with half-strength Hoagland nutrient solution for 3 days and then nitrogen treatment was initiated with modified Hoagland solution containing 0.5 mM NO_3_^-^ (low N supply, Nl), 8 mM NO_3_^-^ (medium N supply, Nm), and 16 mM NO_3_^-^ (high N supply, Nh). Nutrient solutions, aerated to maintain dissolved oxygen, and adjusted to pH of 6.0 with HCl and NaOH, were changed every 3 days. Plants were placed in a climate chamber (*AGC-D001P*, *Qiushi Corp*., China), and the growth conditions were: light intensity of 700 μmol photons m^-2^ s^-1^, a 14 –light period, relative humidity of 60%, and a temperature of 25°C/18°C (day/night).

Polyethylene glycol (PEG 6000) was added to the solutions to act as drought stress until the wheat seedlings had four completely expanded leaves. Different amounts of PEG 6000 (8% PEG and 15% PEG) were added to the nutrient solutions at different nitrogen levels to simulate different drought treatment. The osmotic potential of the solutions were: 0 MPa (well-watered, Ww), -0.15 MPa (moderate water stress, Wm), and -0.4 MPa (severe water stress, Ws).

Our experiments used a completely randomized design with three levels of water potential (0 MPa, -0.15 MPa, and -0.4 MPa; hereafter, Ww, Wm, and Ws) and three levels of nitrogen (0.5 mM, 8 mM, and 16 mM; hereafter, Nl, Nm, and Nh). Each treatment had three replications.

### Plant sampling and measurement

Photosynthetic parameters and Chl *a* fluorescence measurements were measured on fully expanded penultimate leaves of the wheat seedlings between 10:00 h and 11:30 h. Photosynthetic parameters were measured using a Li-6400 gas exchange system *(Li-Cor*, Inc., Lincoln, Nebraska, USA). Intact leaves from each treatment were selected to measure the following variables: photosynthesis rate (*P*_*n*_), transpiration rate (*E*), stomatal conductance (*G*_*s*_), intracellular CO_2_ concentration (*C*_*i*_) and atmospheric CO_2_ concentration (C_a_). The instantaneous water use efficiency (WUE) was calculated as *Pn*/*E*, and the intrinsic water use efficiency (WUE_i_) was determined as *P*_*n*_/*G*_*s*_. The stomatal limitation value (L_s_) was defined as 1-*C*_*i*_/*C*_*a*_ [26]. All photosynthetic measurements were taken at saturating incident photosynthetic photon flux density (PPFD) of 700 μmol photons m^-2^ s^-1^. The temperature was 25 ± 2°C and the concentration of CO_2_ was 380 ± 5 μmol/L.

Chlorophyll *a* fluorescence was measured using a portable chlorophyll fluorometer (FMS 2.02, *Hansatech*, King’s Lynn, UK). After dark-acclimation of leaves that were enclosed in a darkened leaf clip for 30 min, the initial fluorescence (F_o_) was estimated with weak modulated light (<0.1 μmol photons m^-2^ s^-1^), and then leaves was immediately illuminated with an intense saturating flash (>6000 μmol photons m^-2^ s^-1^) to obtain the maximum fluorescence (F_m_). Immediately, the leaves were exposed with an actinic irradiation for 30 min to measure steady state Chl *a* fluorescence (F_s_), saturating pulses(>6000 μmol photons m^-2^ s^-1^) were applied to determine the maximum florescence in the light-adapted state (F_m_’) following each actinic irradiation. Eventually, leaves were illuminated with far-red radiation to determine the minimal fluorescence during the light-adapted state (F_o_’). Other parameters were calculated as follows: PSII maximum photochemical efficiency—F_v_/F_m_ = (Fm—F_o_)/F_m_; excitation energy capture efficiency of PSII reaction centres—F_v_’/F_m_’ = (F_m_’- F_o_’)/F_m_’; the quantum yield of PSII— Φ_PSII_ = (F_m_’- F_s_)/F_m_’; photochemical quenching—qP = (F_m_’—F_s_) /(F_m_’- F_o_’); non-photochemical quenching—NPQ = (Fm—F_m_’)/F_m_’; the apparent photosynthetic electron transport rate (ETR) was calculated as PPFD×0.84×0.5×Φ_PSII_ [14, 27].

After the drought treatment to the wheat seedlings for 7 days, they were cut into shoot and root portions and their surfaces were rinsed with water; after this, these samples were placed in a drying oven to inactivate the enzymes at 90°C for 30 min and then dried at 75°C to obtain the dry matter. Finally the shoot dry mass (SM) and root dry mass (RM) was determined.

### Statistical analysis

All data was submitted to ANOVA test using the General Linear Model procedure and the differences between the means of treatments were compared using Duncan’s multiple range test (*P<0*.*05*). Pearson linear correlation (*P<0*.*05*) was performed to test significance between the parameter correlations. All data were processed using SPSS 19.0 software for windows (SPSS Inc., Chicago, IL, USA).

## Results

### Photosynthetic characteristics with different nitrogen supply and water regimes

Effects of nitrogen levels on *P*_*n*_, *E*, *G*_*s*_, and *C*_*i*_ were not identical under different water conditions (Table 1). With drought stress, *P*_*n*_, *E*, and *G*_*s*_ decreased significantly (*P*<0.01), meanwhile WUE and WUE_i_ remarkably improved at all N treatments in both the cultivars. *C*_*i*_ decreased slightly, and L_s_ increased with increasing water stress in the low N and medium N supply treatments.

**Table 1:** Interactive effects of N supply and water status on gas exchange and water use efficiency in two varieties of wheat.

Treatment	*P*_*n*_ [μmol (CO_2_ m^-2^s^-1^)]		*E* [mmol (H_2_O m^-2^s^-1^)]		*G*_*s*_ [mol (H_2_O m^-2^s^-1^)]		*C*_*i*_ [μmol (CO_2_) mol^-1^]		WUE [μmol(CO_2_)mol(H_2_O)^-1^]		WUE_i_ [μmol(CO_2_)mol(H_2_O)^-1^]		Ls	
	Xinong979	Large-spike	Xinong979	Large-spike	Xinong979	Large-spike	Xinong979	Large-spike	Xinong979	Large-spike	Xinong979	Large-spike	Xinong979	Large-spike
NlWw	17.26±0.22^cd^	16.14±0.38^d^	7.72±0.19^e^	6.64±0.21^e^	0.437±0.01^d^	0.521±0.01^d^	258.0±1.08^e^	250.8±1.05^g^	2.25±0.07^a^	2.44±0.06^bc^	39.49±0.69^a^	30.98±0.66^a^	0.330±0.01^a^	0.351±0.01^a^
NlWm	13.67±0.36^b^	11.44±0.38^b^	4.35±0.21^c^	6.89±0.14^e^	0.229±0.01^b^	0.381±0.01^c^	236.1±1.75^b^	230.2±0.87^c^	3.21±0.20^b^	1.67±0.08^a^	60.55±3.37^c^	30.15±1.26^a^	0.389±0.01^d^	0.402±0.01^d^
NlWs	6.96±0.39^a^	6.86±0.41^a^	1.16±0.07^a^	2.97±0.13^ab^	0.125±0.00^a^	0.162±0.01^a^	230.3±1.12^b^	219.0±1.06^a^	6.17±0.49^d^	2.33±0.17^b^	56.37±4.59^c^	43.46±3.81^bc^	0.400±0.01^e^	0.436±0.01^f^
NmWw	18.23±0.18^d^	17.65±0.49^d^	8.15±0.24^e^	7.85±0.16^f^	0.558±0.01^e^	0.578±0.01^e^	243.1±1.28^c^	241.8±0.81^ef^	2.25±0.07^a^	2.26±0.07^b^	32.77±0.85^a^	30.67±1.26^a^	0.365±0.01^c^	0.375±0.01^bc^
NmWm	17.30±0.20^cd^	14.14±0.59^c^	7.24±0.31^e^	4.16±0.20^d^	0.400±0.01^d^	0.261±0.01^b^	234.0±1.17^b^	219.4±1.91^a^	2.43±0.12^ab^	3.46±0.20^de^	43.84±2.06a^ab^	54.45±2.71^de^	0.388±0.01^d^	0.429±0.01^ef^
NmWs	13.76±0.25^b^	11.55±0.46^b^	3.41±0.17^b^	3.44±0.168^c^	0.147±0.01^a^	0.185±0.01^a^	212.7±2.19^a^	225.4±1.44^bc^	4.10±0.17^c^	3.41±0.21^de^	95.68±4.77^d^	63.08±3.16^e^	0.448±0.01^f^	0.417±0.01^e^
NhWw	18.22±0.18^d^	17.82±0.39^d^	7.89±0.23^e^	6.88±0.24^e^	0.537±0.01^e^	0.493±0.01^d^	236.3±1.20^b^	237.6±1.11^d^	2.33±0.07^ab^	2.61±0.07^bc^	34.09±1.05^a^	36.20±0.86^ab^	0.386±0.01^d^	0.386±0.01^c^
NhWm	16.76±0.27^c^	12.03±0.48^b^	5.57±0.25^d^	4.04±0.10^cd^	0.311±0.01^c^	0.249±0.01^b^	250.1±0.75^d^	243.3±1.01^f^	3.06±0.15^ab^	2.98±0.12^cd^	54.36±1.91^bc^	48.67±2.32^cd^	0.346±0.01^b^	0.368±0.01^b^
NhWs	14.21±0.23^b^	10.40±0.45^b^	5.52±0.24^d^	2.69±0.14^a^	0.281±0.01^c^	0.171±0.01^a^	243.7±1.82^cd^	224.3±1.00^ab^	2.62±0.14^ab^	3.91±0.13^e^	51.37±2.86^bc^	61.87±4.32^e^	0.360±0.01^c^	0.417±0.01^e^
C	***		***		ns		***		***		***		***	
N	***		***		***		***		ns		***		***	
W	***		***		***		***		***		***		***	
C×N	***		***		***		***		***		***		***	
C×W	***		ns		**		**		***		*		***	
N×W	***		***		***		***		***		***		***	
C×W×N	**		***		***		***		***		***		***	

*P*_*n*_*—*net photosynthetic rate; *E–*transpiration rate, *G*_*s*_*—*stomatal conductance; *C*_*i*_*—*intercellular CO_2_ concentration; WUE—instantaneous water use efficiency; WUE_i_—intrinsic water use efficiency; L_s_—stomatal limitation value (based on [9]); C—cultivars; N—nitrogen; W—water. Nl—low N; Nm—medium N; Nh—high N; Ww—well-watered; Wm—moderate water stress; Ws—severe water stress. Values are means± standard error (SE), *n* = 3. Different letters within the same column denote the differences between the treatments (*P*<0.05). NS: no significant at the 0.05 level; *, **, ***, significant at *P*<0.05, *P*<0.01 and *P*<0.001, respectively.

The *P*_*n*_ and *E* of the two cultivars were consistent under well-watered conditions for all N treatments. Low N treatment had a smaller *P*_*n*_ than the medium N and high N treatments under drought stress, but no significant difference was found between the medium N and high N treatments. The *P*_*n*_ in the low N treatments decreased markedly under severe water stress while that of the plants in the high N treatment decreased slowly under all water regimes. Under the moderate water stress treatments, the *G*_*s*_ of Xinong 979 under the low N treatment significantly decreased compared with other N supply treatments, whereas it increased in large-spike wheat. Under the severe water stress treatments, the high N treatment increased *G*_*s*_ more than the low N and medium N treatments for Xinong 979, but no significant difference was observed in large-spike wheat. There was no significant difference between WUE and WUE_i_ under well-watered conditions of the plants at any level of nitrogen. *C*_*i*_ decreased and L_s_ increased with increasing nitrogen concentrations under well-watered conditions.

Under water stress condition, Xinong 979 showed higher values of *P*_*n*_ than large-spike wheat in all N treatments, and it also showed higher values of *E* than large-spike wheat except under the low N treatments under water stress. Xinong 979 under the low N and medium N treatments and large-spike wheat under the high N treatment had a significantly improved value of WUE with increased drought. Under water-stressed conditions, large-spike wheat had a higher L_s_ compared with Xinong 979 under the low N and high N treatments.

The three-way *ANOVA* (Table 1) revealed that there was a significant difference among *P*_*n*_, *E*, *C*_*i*_, WUE_i_ and L_s_ under different water conditions, nitrogen levels and cultivars. Moreover, they showed significant two-way interactions on *P*_*n*_, *G*_*s*_, *C*_*i*_, WUE, WUE_i_, and L_s_ between the cultivars, nitrogen and water. Also a significant three-way interaction was observed among these factors. No significant differences were found in *G*_*s*_ between cultivars and in WUE between nitrogen, but there were significant interactions among cultivars, nitrogen, and water.

### Chl *a* fluorescence parameters with different nitrogen supply and water regime

Water and nitrogen coupling had significant effects on fluorescence parameters of wheat (Table 2). For both cultivars, significant effects were observed under water stress in F_v_/F_m_, F_v_/F_o_, Φ_PSII_ and ETR, and the parameters decreased markedly under water stress at all N treatments. qP decreased slightly with increasing drought while NPQ increased. F_m_ decreased significantly under the severe water stress treatment and for large-spike wheat under the low N treatment. No significant difference was found for F_o_ under all water conditions in Xinong 979, but it increased with increasing water stress for large-spike wheat.

**Table 2:** Interactive effects of N supply and water status on Chl *a* fluorescence parameters in two varieties of wheat.

Treatment	F_o_		F_m_		qP		NPQ		F_v_/F_m_		F_v_/F_o_		Φ_PSII_		ETR	
	Xinong979	Large-spike	Xinong979	Large-spike	Xinong979	Large-spike	Xinong979	Large-spike	Xinong979	Large-spike	Xinong979	Large-spike	Xinong979	Large-spike	Xinong979	Large-spike
NlWw	141.8±0.83^a^	122.5±1.50^a^	746.0±3.68^c^	695.3±3.46^a^	0.911±0.01^cde^	0.840±0.01^c^	0.418±0.01^b^	0.396±0.01^a^	0.810±0.01^c^	0.824±0.01^c^	4.26±0.05^b^	4.68±0.09^c^	0.691±0.01^b^	0.675±0.01^c^	0.964±0.01^bc^	1.425±0.02^c^
NlWm	139.3±2.81^a^	143.3±6.79^b^	683.5±14.73^b^	680.0±8.83^a^	0.894±0.01^bcd^	0.786±0.01^b^	0.427±0.01^b^	0.445±0.01^b^	0.796±0.01^b^	0.789±0.01^b^	3.91±0.07^b^	3.77±0.26^b^	0.674±0.01^b^	0.644±0.01^b^	0.841±0.02^ab^	1.257±0.01^b^
NlWs	140.0±0.76^a^	168.2±1.01^c^	630.0±1.52^a^	697.2±3.81^a^	0.851±0.01^a^	0.749±0.01^a^	0.316±0.01^a^	0.482±0.01^c^	0.777±0.01^a^	0.759±0.01^a^	3.50±0.03^a^	3.14±0.02^a^	0.640±0.01^a^	0.600±0.01^a^	0.733±0.01^a^	1.069±0.02^a^
NmWw	160.8±4.04^b^	148.7±2.02^b^	1014.0±9.98^e^	980.5±4.72^c^	0.929±0.01^e^	0.918±0.01^fg^	0.639±0.01^c^	0.706±0.01^d^	0.841±0.01^e^	0.848±0.01^d^	5.31±0.11^d^	5.600±0.11^e^	0.716±0.01^c^	0.718±0.01^e^	1.579±0.01^ef^	1.744±0.02^ef^
NmWm	160.0±4.61^b^	155.2±3.34^bc^	943.8±5.94^d^	980.0±5.56^c^	0.920±0.01^de^	0.884±0.01^d^	0.689±0.01^d^	0.728±0.01^d^	0.830±0.01^de^	0.842±0.01^cd^	4.91±0.16^cd^	5.32±0.16^de^	0.717±0.01^c^	0.691±0.01^cd^	1.317±0.05^d^	1.672±0.03^e^
NmWs	165.2±2.33^b^	167.2±2.77^c^	943.0±9.92^d^	926.5±2.46^b^	0.898±0.01^bcd^	0.891±0.01^de^	0.715±0.01^de^	0.720±0.01^d^	0.825±0.01^d^	0.820±0.01^c^	4.71±0.13^c^	4.55±0.09^c^	0.681±0.01^b^	0.667±0.01^bc^	1.056±0.06^c^	1.583±0.01^d^
NhWw	158.8±2.74^b^	155.3±2.90^bc^	993.7±2.74^e^	979.3±5.04^c^	0.889±0.01^bc^	0.926±0.01^g^	0.734±0.01^de^	0.733±0.01^d^	0.840±0.01^e^	0.841±0.01^cd^	5.26±0.12^d^	5.31±0.15^de^	0.721±0.01^c^	0.711±0.01^de^	1.739±0.04^g^	1.786±0.01^f^
NhWm	163.5±1.75^b^	166.0±2.08^c^	994.5±4.09^e^	988.0±4.00^c^	0.871±0.01^ab^	0.910±0.01^efg^	0.745±0.01^e^	0.722±0.01^d^	0.836±0.01^de^	0.832±0.01^cd^	5.08±0.06^cd^	4.95±0.05^cd^	0.675±0.01^b^	0.675±0.01^c^	1.703±0.02^fg^	1.546±0.01^d^
NhWs	161.2.±1.20^b^	167.0±3.21^c^	946.2±4.49^d^	937.0±5.85^b^	0.878±0.01^b^	0.899±0.01^def^	0.708±0.01^de^	0.746±0.01^d^	0.830±0.01^de^	0.822±0.01^c^	4.87±0.04^cd^	4.61±0.09^c^	0.669±0.01^b^	0.684±0.01^c^	1.534±0.04^e^	1.519±0.01^d^
C	ns		ns		***		***		ns		ns		***		***	
N	***		***		***		***		***		***		***		***	
W	***		***		***		**		***		***		***		***	
C×N	*		ns		***		**		ns		ns		***		***	
C×W	***		***		*		***		**		**		ns		ns	
N×W	**		***		***		**		***		*		***		ns	
C×W×N	**		***		*		***		ns		ns		*		***	

F_0_—minimal fluorescence of dark-adapted state; F_m_—maximal fluorescence of dark-adapted state; F_v_—variable fluorescence; qP-photochemical quenching coefficient; NPQ—non-photochemical quenching; F_v_/F_m_—maximum quantum yield of PS II photochemistry; F_v_/F_0_ –maximum energy transformation potential of PS II photochemistry; Φ_PSII_—effective quantum yield of PS II photochemistry; ETR—electron transport rate (based on [14, 27]); C—cultivars; N—nitrogen; W—water. Nl—low N; Nm—medium N; Nh—high N; Ww—well-watered; Wm—moderate water stress; Ws—severe water stress. Values are means± standard deviation (SD), *n* = 3. Different letters within the same column denote the differences between the treatments (*P*<0.05). NS: no significant at the 0.05 level; *, **, ***, significant at *P*<0.05, *P*<0.01 and *P*<0.001, respectively.

The low N treatment decreased F_o_, F_m_, qP, NPQ, F_v_/F_m_, F_v_/F_o_, Φ_PSII_, and ETR markedly decreased compared with the medium N and high N treatments for both cultivars (Table 3). No significant difference was observed in F_o_, F_m_, F_v_/F_m_, F_v_/F_o_, and Φ_PSII_ between the low N and high N treatments. The wheat in the high N treatment had a higher qP and NPQ than in the medium N treatment. ETR increased with increases in the nitrogen concentration in Xinong 979 regardless of water conditions, whereas it decreased under water stress for the high N treatment in large-spike wheat.

**Table 3:** Dry mass partitioning of two wheat cultivars under different nitrogen levels and water status.

Treatment	RM [g (DM) plant^-1^]		SM [g (DM) plant^-1^]		TDM [g (DM) plant^-1^]		R/S [g g^-1^]	
	Xinong979	Large-spike	Xinong979	Large-spike	Xinong979	Large-spike	Xinong979	Large-spike
NlWw	0.114±0.01^a^	0.114±0.02^a^	0.104±0.01^a^	0.111±0.01^a^	0.218±0.01^a^	0.226±0.04^a^	1.103±0.08^b^	1.027±0.13^a^
NlWm	0.125±0.01^ab^	0.122±0.01^a^	0.103±0.01^a^	0.110±0.01^a^	0.229±0.01^a^	0.232±0.01^a^	1.216±0.11^bc^	1.120±0.17^a^
NlWs	0.130±0.01^ab^	0.110±0.01^a^	0.097±0.01^a^	0.095±0.01^a^	0.226±0.02^a^	0.205±0.01^a^	1.352±0.12^c^	1.181±0.26^a^
NmWw	0.203±0.01^d^	0.205±0.01^d^	0.555±0.01^d^	0.431±0.02^c^	0.757±0.01^d^	0.637±0.03^c^	0.366±0.01^a^	0.475±0.13^b^
NmWm	0.163±0.01^c^	0.156±0.01^bc^	0.427±0.02^c^	0.349±0.02^b^	0.590±0.03^c^	0.505±0.02^b^	0.382±0.02^a^	0.447±0.02^b^
NmWs	0.166±0.01^c^	0.178±0.01^cd^	0.423±0.02^c^	0.305±0.02^b^	0.589±0.02^c^	0.482±0.03^b^	0.393±0.01^a^	0.583±0.01^b^
NhWw	0.161±0.01^c^	0.142±0.01^ab^	0.392±0.01^bc^	0.329±0.02^b^	0.552±0.01^c^	0.471±0.03^b^	0.410±0.02^a^	0.432±0.02^b^
NhWm	0.131±0.01^ab^	0.171±0.01^bc^	0.365±0.02^b^	0.492±0.04^d^	0.495±0.01^b^	0.663±0.05^c^	0.359±0.03^a^	0.348±0.01^b^
NhWs	0.136±0.01^b^	0.177±0.01^cd^	0.349±0.04^b^	0.325±0.02^b^	0.485±0.04^b^	0.502±0.03^b^	0.394±0.04^a^	0.544±0.02^b^

RM—root dry mass; SM—shoot dry mass; TDM—total dry mass; R/S—root—shoot ratio. Nl—low N; Nm—medium N; Nh—high N; Ww—well-watered; Wm—moderate water stress; Ws—severe water stress. Values are means± standard error (SE), *n* = 3. Different letters within the same column denote the differences between the treatments (*P*<0.05).

Xinong 979 had a higher qP and Φ_PSII_ than large-spike wheat for the low N treatment under all water conditions. The ETR in Xinong 979 was smaller than in large-spike wheat for the low N and medium N treatments. No significant difference was found in F_o_, F_m_, F_v_/F_m_, and F_v_/F_o_ between the two cultivars (Table 2).

The three-way *ANOVA* analysis (Table 2) proved that there was significant (*p<0*.*05*) effect of nitrogen supply and water conditions on all fluorescence parameters, and they also showed, except for ETR, significant two-way interactions (*p*<0.05) with water and nitrogen. No significant differences were observed on F_o_, F_m_, F_v_/F_m_, F_v_/F_o_ between the cultivars. There were significant effects on qP and NPQ as a dependent variable and interactions among the cultivars, nitrogen and water.

### Dry mass partitioning with different nitrogen levels and water regimes

The root dry mass (RM), shoot dry mass (SM), total dry mass (TDM) and the root—shoot ratio (R/S) showed a significant effect when there was an interaction between the nitrogen supply and water conditions. As shown in Table 3, plants of both the cultivars under the low N treatment had a significantly smaller root dry mass, shoot dry mass, and total dry mass, but a higher root—shoot ratio compared with medium N and high N for all water regimes. Under adequate water conditions, wheat plants in the medium N treatment had a higher root dry mass, shoot dry mass, and total dry mass compared with the high N treatment in both the cultivars. Water stress under all nitrogen supply treatments increased the root—shoot ratioslightly, but no significant differences were found between medium N and high N treatments under all water regimes.

For both cultivars, water stress in the low N treatment had no significant impact on root dry mass, shoot dry mass, total dry mass and root—shoot ratio. Under the medium N and high N treatment, both water stress decreased root dry mass, shoot dry mass, and total dry mass, and no significant differences were found in root dry mass, shoot dry mass, and total dry mass between the moderate water stress and severe water stress treatments. However, large-spike wheat had a higher shoot dry mass and total dry mass under moderate water stress treatment compared with severe water stress treatment.

Xinong 979 had a higher shoot dry mass and total dry mass and smaller root—shoot ratio compared with large-spike wheat under the medium N treatment. Large-spike wheat had a higher root dry mass, shoot dry mass, and total dry mass compared with Xinong 979 in the high N treatment under moderate water stress.

### Relationships between traits and multivariate analysis

A multiple correlation analysis was performed between different gas-exchange characteristics and plant biomass (Table 4). For both varieties, the total dry mass (TDM) showed significant positive correlation with *P*_*n*_, qP, NPQ, F_v_/F_m_, F_v_/F_o_, Φ_PSII_, and ETR and significant negative correlation with root—shoot ratio. For large-spike wheat, the total dry mass showed a positive correlation with WUE and WUE_i_ and no correlation with *G*_*s*_ and *E*. For Xinong 979, the total dry mass did not show any correlation with WUE and WUE_i_ but showed a positive correlation with *G*_*s*_ and *E*. Likewise, for both the varieties, the root—shoot ratio (R/S) showed significant negative correlation with *P*_*n*_, NPQ, F_v_/F_m_, F_v_/F_o_, Φ_PSII_, and ETR. The total dry mass in large-spike wheat showed a significant negative correlation with qP, whereas there was no correlation in Xinong 979.

**Table 4:** Pearson’s correlation coefficients among the biomass, gas exchange and Chl *a* fluorescence parameters in two varieties of wheat.

parameter	TDM	RSR	*P*_*n*_	*G*_*s*_	*C*_*i*_	*E*	WUE	WUE_i_	L_s_	qP	NPQ	F_v_/F_m_	F_v_/F_o_	Φ_PSII_	ETR
TDM	1	-0.899**	0.573**	0.398*	-0.188	0.405*	-0.356	0.030	0.150	0.454*	0.809**	0.826**	0.838**	0.632**	0.717**
RSR	-0.888**	1	-0.652**	-0.348	0.066	-0.418*	-0.437*	-0.084	-0.105	-0.324	-0.962**	-0.909**	-0.900**	-0.553**	-0.833**
*P*_*n*_	0.389*	-0.430*	1	0.795**	0.428*	0.880**	-0.775**	-0.347	-0.431*	0.638**	0.614**	0.814**	0.794**	0.789**	0.692**
*G*_*s*_	0.009	0.000	0.762**	1	0.591**	0.908**	-0.679**	-0.762**	-0.553**	0.570**	0.296	0.600**	0.609**	0.786**	0.600**
*C*_*i*_	0.171	-0.138	0.672**	0.755**	1	0.586**	-0.410*	-0.714**	-0.984*	0.103	-0.109	0.174	0.173	0.141	0.320
*E*	-0.004	0.050	0.704**	0.941**	0.675**	1	-0.868**	-0.584**	-0.572*	-0.681**	0.378	0.669**	0.648**	0.799**	0.548**
WUE	0.458*	-0.552**	0.046	-0.527**	-0.313	-0.634**	1	0.405*	0.422*	-0.653**	-0.475*	-0.657**	-0.611**	-0.700**	-0.497**
WUE_i_	0.415*	-0.429*	-0.123	-0.690**	-0.402*	-0.656**	0.855**	1	0.683*	-0.245	0.111	-0.169	-0.193	-0.390*	-0.331
L_s_	-0.180	0.150	-0.690**	-0.772**	-0.991**	-0.684**	0.299	0.417*	1	-0.122	-0.058	-0.207	-0.202	-0.145	-0.345
qP	0.853**	-0.900**	0.654**	0.218	0.344	0.142	0.514**	0.374	-0.355	1	0.210	0.447*	0.420*	0.754**	0.151
NPQ	0.873**	-0.890**	0.257	-0.221	-0.156	-0.247	0.652**	0.586**	0.149	0.829**	1	0.895**	0.879**	0.500**	0.832**
F_v_/F_m_	0.721**	-0.789**	0.799**	0.438*	0.452*	0.349	0.388*	0.189	-0.471*	0.903**	0.626**	1	0.995**	0.716**	0.743**
F_v_/F_o_	0.727**	-0.784**	0.802**	0.474*	0.446*	0.377	0.331	0.126	-0.466*	0.884**	0.633**	0.989**	1	0.713**	0.892**
Φ_PSII_	0.673**	-0.742**	0.820**	0.542**	0.481*	0.456*	0.283	0.054	-0.495**	0.883**	0.606**	0.927**	0.921**	1	0.543**
ETR	0.763**	-0.832**	0.768**	0.373	0.347	0.303	0.407*	0.246	-0.363	0.931**	0.753**	0.931**	0.935**	0.927**	1

TDM—total dry mass; R/S—root—shoot ratio; *P*_*n*_*—*net photosynthetic rate; *E–*transpiration rate, *G*_*s*_*—*stomatal conductance; *C*_*i*_*—*intercellular CO_2_ concentration; WUE—instantaneous water use efficiency; WUE_i_—intrinsic water use efficiency; L_s_—stomatal limitation value; qP-photochemical quenching coefficient; NPQ—non-photochemical quenching; F_v_/F_m_—maximum quantum yield of PS II photochemistry; F_v_/F_0_—maximum energy transformation potential of PS II photochemistry; Φ_PSII_—effective quantum yield of PS II photochemistry; ETR—electron transport rate. The correlations were calculated using the means of all water and nitrogen levels (*n* = 9). The upper and lower triangle denotes Xinong 979 and large-spike wheat, respectively. Probabilities (* *P*≦0.05; ** *P*≦0.01) are shown.

For both varieties, *P*_*n*_ showed significant positive correlation with *G*_*s*_, *C*_*i*_, *E*, qP, F_v_/F_m_ F_v_/F_o_, Φ_PSII_, and ETR and significant negative correlation with L_s_. The *P*_*n*_ in large-spike wheat did not show a significant correlation with WUE, WUE_i_ and NPQ. In contrast, the *P*_*n*_ in Xinong 979 showed a negative correlation with WUE, while there was a significant positive correlation with NPQ. For both cultivars, *G*_*s*_ showed a significant positive correlation with *C*_*i*_, *E*, F_v_/F_m_, F_v_/F_o_, and Φ_PSII_ and a negative correlation with WUE, WUE_i_, and L_s_. For large-spike wheat, *G*_*s*_ showed no correlation with qP and ETR, but there was a positive correlation between them in Xinong 979. *E* was negatively correlated with WUE, WUE_i_ and L_s_ in both the cultivars. No correlation was detected between *E* and qP, NPQ, F_v_/F_m_, F_V_/F_o_, or ETR in large-spike wheat. In contrast, *E* in Xinong 979 showed a significant positive correlation with qP, F_v_/F_m_, F_v_/F_o_, Φ_PSII_ and ETR.

For large-spike wheat, WUE showed significant positive correlation with WUE_i_, qP, and NPQ and no correlation with F_v_/F_o_, or Φ_PSII_. For Xinong 979, WUE showed positive correlation with WUE_i_ and L_s_. Moreover, WUE showed significant negative correlation with qP, NPQ, F_v_/F_m_, Φ_PSII_, and ETR. There was significant or extremely significant positive correlation between qP, NPQ, F_v_/F_m_, F_v_/F_o_, Φ_PSII_, and ETR in both the cultivars, but no correlation was found between qP and NPQ or ETR in Xinong 979.

## Discussion

Water and nitrogen are the most basic environmental factors that affect the growth of wheat, and their effects on the ecological environment of crops are not isolated but rather mutually influenced and restricted. The effects of drought stress and nitrogen levels on the physiological mechanisms of wheat have been investigated in many previous studies [3, 28]. Water and nitrogen deficiency can inhibit stomatal opening and photosynthesis. Improving wheat yield using the beneficial management of water and nitrogen supply is an important strategy.

It is well known that *P*_*n*_ can be affected by stomatal and nonstomatal factors [29]. It is generally believed that the decrease of stomatal aperture and increase of stomatal resistance can cause lower *G*_*s*,_ resulting in prevention of carbon fixation, and eventually this contributes to depressed photosynthetic rates [10]. In our experiment, the performance of reduction on *P*_*n*_, *E*, and *G*_*s*_ differed under different N conditions after the imposition of drought stress between the two cultivars. Under the low N and medium N conditions, the large decrease in *G*_*s*_ and *C*_*i*_ with increased water stress suggests that stomatal closure induced by water stress, which influences the diffusion of CO_2_ from the atmosphere to the cell interior [30], is the major reason for the decline of *P*_*n*_. A significant positive correlation was observed between the *G*_*s*_ and *P*_*n*_ at all measurements, also supporting this suggestion. Thus, the most important limiting factor of photosynthesis in the low and moderate nitrogen treatment, in contrast with the high nitrogen treatment is stomatal closure regardless of any moisture conditions.

In contrast, WUE and WUE_i_ improved with stress. In the case of the stomatal limitation becoming the dominant factor in photosynthesis, the *P*_*n*_ increased and the WUE improved accordingly by reducing transpiration loss due to stomatal limitation. *P*_*n*_ showed a positive correlation with leaf WUE, which is also the mechanism of improving crop WUE through stomatal control.

In the high N treatment, the decline in *P*_*n*_ under drought stress was not associated with stomatal closure, but was caused by non-stomatal limitation because of the decline of photosynthetic activity of mesophyll. This may have been due to the change of chloroplast structure and the damage of membrane system, followed by damage of the photosynthetic electron transport system; further, the synthase activity may have decreased and hydrolase activity increased [9], finally leading to the decline of carboxylation efficiency. The decrease of L_s_ and increase of *C*_*i*_ indicate that non-stomatal limitation prevailed for the decline of *P*_*n*_ under high N conditions [31]. Nitrogen affects physiological characteristics of plants through regulation of photosynthesis, transpiration and respiration [13]. In this study, the increase in *P*_*n*_ at high N supply may be due to the N supply improving the activity of photosynthetic apparatus and carboxylation efficiency in leaves. Similar results were observed in other plants, such as *Prunella vulgaris* [32] and *Sophora davidii* seedlings [33]. When water is sufficient, the excessive nitrogen supply did not improve the photosynthetic rate of wheat significantly. Under water stress, the *G*_*s*_ decreased faster than the *P*_*n*_ and *E* decreased due to stomatal closure, which caused an increase in WUE and WUE_i_. Our findings are in agreement with the findings in other crops under a water deficit [34] and salinity stress [35].

The difference in the Chl *a* fluorescence parameters observed in different varieties is possibly due to their different response to nitrogen fertilizer and the interaction of other environmental conditions and nitrogen fertilizers. In current studies, we have observed that Chl *a* fluorescence parameters show significant differences with different N application levels. Nitrogen deficiency usually led to a decline in protein synthesis, resulting in photodamage to the PSII reaction center which could not be restored effectively and, thus, photoinhibition. The decrease of F_v_/F_m_ is a remarkable characteristic of photoinhibition [36]. The decrease of F_v_/F_m_ is likely to be due to the inactivation of PSIIactivity and a decrease in the transfer of excitation energy from the light-harvesting complex (LHCII) to PSII, which may be related to the decrease of LHCII content under water deficit [15]. In our experiments, the low N level led to significant reduction in F_m_, F_V_/F_m_ and F_v_/F_o_, which might inhibit photochemical activities and potential photosynthesis activity in PSII. Meanwhile, the decline of ETR led to the generation of excess excitation energy, which in turn aggravated photoinhibition under drought conditions [37]. For Large-spike wheat, F_o_ significantly increased under water stress, suggesting that drought stress damages PSII reaction centers and inhibits primary photochemical reactions [38].

In our study, we observed that the Φ_PSII_ parameters decreased under drought stress but improved with nitrogen supply, indicating that excitation energy captured by PSII reaction centers and the energy used for photochemical capacity may have declined under water deficit and that the added nitrogen might have promoted photochemical activity of PSII [39]. Similar effects have been observed in *Sophora davidll* seedlings [33] responding to water stress and sugar beets responding to addition of nitrogen [40]. In the two cultivars used here, water stress and N deficiency induced a significant reduction of Φ_PSII_, indicating a decline in the electron transport activities in PSII. The increased excitation energy seems to have dissipated in the form of heat (NPQ) to protect the photosynthetic apparatus from damage [41, 42]. qP represents the fraction of light energy captured by the antenna pigments in PSII, used for photochemical electron transfer [43]. NPQ represents the fraction of light energy that cannot be used to photochemical electron transfer, but dissipated in the form of heat, as noted above [44]. In our study, qP showed an effect similar to that of with Φ_PSII_ in response to drought stress. NPQ increased with increase in nitrogen concentration, while no significant effect was found for water stress. These data suggest that nitrogen deficiency can lead to reduction of photochemical efficiency in wheat leaves under water stress. Furthermore, an excess of N supply is not conducive to the utilization of trapped light energy effectively.

Under drought stress, initial Chl fluorescence (F_o_) increased significantly, the maximum photochemical efficiency (F_v_/F_m_) and photochemical quenching coefficient (qP) decreased resulting in the decline of the quantum yield of PSII (Φ_PSII_) [45, 46]. Moreover, low electron transport through PSII and the loss of PSII activity (F_v_/F_o_) eventually result in a decrease in net photosynthetic rate (*P*_*n*_) [37]. Nitrogen supply can improve photosynthetic performance by maintaining F_v_/F_m_ and F_v_/F_o_ at a relatively high level and improving the apparent photosynthetic electron transport rate (ETR) and Φ_PSII_, resulting in efficient conversion of light energy into a usable chemical energy for photosynthesis [47].

The root—shoot ratio (R/S) is a parameter that reflects a response of root and shoot growth to various environmental conditions [21]. Previous studies have shown that R/S increases under water deficit [48, 49] or under nitrogen deficiency [19, 50]. In this study, the increase in R/S under low N supply was mainly because of the declining shoot biomass more than the decrease in the root biomass. Nitrogen deficiency lead to the increase of root—shoot ratio due to a restrain in the shoot growth, and that excessive nitrogen supply lead to the reduction of root—shoot ratio due to restraining of growth of root and improved shoot growth [51]. In the present study, the root—shoot ratio of the two varieties between moderate N and high N supply under water stress were not consistent; however, they all showed the smallest root—shoot ratio under high N supply and moderate water stress treatment. There were significant effects of nitrogen levels on root—shoot ratio, and root—shoot ratio did not exhibit significant responses to the interactive of nitrogen and water.

Our study clearly showed that nitrogen deficiency produced a significant effect on Chl *a* fluorescence. Xinong 979 with a higher *P*_*n*_, qP, Φ_PSII_, F_v_/F_m_, F_v_/F_o_ than large-spike wheat indicated better acclimation, in the former, under a nitrogen deficiency. For large-spike wheat, *P*_*n*_ decreased faster under water stress at high N levels than Xinong 979, suggesting that excessive nitrogen may have increased plant drought stress. The root biomass decreased in Xinong 979 and increased in large-spike wheat under water stress but with high N supply treatment. This indicates that the nitrogen demand of plant growth was different in different cultivars under water stress. Xinong 979 with drought tolerance was highly resistant to environmental changes. Under water stress and high nitrogen supply, there were small variations of stomatal conductance and transpiration in Xinong 979, and the water use efficiency was high. In comparison, the large-spike wheat with drought sensitivity, was revealed to possess highly fluctuating photosynthetic physiological indexes under changing water and nitrogen condition.

### Conclusions

In conclusion, the response to photosynthetic parameters in different wheat varieties were probably different even under the same water and fertilizer conditions, which related to other characteristics of the two wheat varieties used. The decreases in *P*_*n*_ under water deficit may be due to the closure of stomata caused by osmotic stress or else by the damage of photosynthetic apparatus and other metabolic processes caused by drought. Water stress not only results in the decrease of photosynthesis and transpiration rates but also affects the efficiency of PSII; In fact, the latter may be one of the causes of changes in photosynthesis. Appropriate increase of the nitrogen supply can improve energy conversion efficiency and potential activity of PSII; also, there may be enhancement in excess light energy dissipation, which may reduce inhibition of photosynthesis under environmental stress and enhance the stability of the photosynthetic reaction center, and provide sufficient energy for carbon assimilation. Thus the photosynthesis capacity and biomass of wheat were improved effectively. Under severe water stress, nitrogen application showed a negative effect on photosynthesis. An excessive nitrogen supply had no effect on plant drought resistance, and it even had an adverse effect on plant growth. Thus, an appropriate nitrogen supply may be conducive in enhancing drought resistance of wheat by improving the photosynthesis processes and decreasing the injury of photosynthetic apparatus under water deficit. Xinong 979 has a higher photosynthetic rate and biomass under drought-stressed and N deficiency conditions when compared to Large-spike wheat. The result suggests that Xinong 979 has a stronger drought resistance compared with large-spike wheat under N deficiency.
